# An improved algorithm based on YOLOv5 for detecting Ambrosia trifida in UAV images

**DOI:** 10.3389/fpls.2024.1360419

**Published:** 2024-05-10

**Authors:** Chen Xiaoming, Chen Tianzeng, Meng Haomin, Zhang Ziqi, Wang Dehua, Sun Jianchao, Wang Jun

**Affiliations:** College of Engineering and Technology, Jilin Agricultural University, Changchun, China

**Keywords:** deep learning, unmanned aerial vehicle, small object detection, YOLOv5, invasive plant

## Abstract

A YOLOv5-based YOLOv5-KE unmanned aerial vehicle (UAV) image detection algorithm is proposed to address the low detection accuracy caused by the small size, high density, and overlapping leaves of Ambrosia trifida targets in UAV images. The YOLOv5-KE algorithm builds upon the YOLOv5 algorithm by adding a micro-scale detection layer, adjusting the hierarchical detection settings based on k-Means for Anchor Box, improving the loss function of CIoU, reselecting and improving the detection box fusion algorithm. Comparative validation experiments of the YOLOv5-KE algorithm for Ambrosia trifida recognition were conducted using a self-built dataset. The experimental results show that the best detection accuracy of Ambrosia trifida in UAV images is 93.9%, which is 15.2% higher than the original YOLOv5. Furthermore, this algorithm also outperforms other existing object detection algorithms such as YOLOv7, DC-YOLOv8, YOLO-NAS, RT-DETR, Faster RCNN, SSD, and Retina Net. Therefore, YOLOv5-KE is a practical algorithm for detecting Ambrosia trifida under complex field conditions. This algorithm shows good potential in detecting weeds of small, high-density, and overlapping leafy targets in UAV images, it could provide technical reference for the detection of similar plants.

## 1 Introduction

Deep learning (DL) is a powerful machine-learning technique that achieves state-of-the-art results in various tasks such as image classification (Haq et al., 2021b; Haq, 2022a; Gulzar, 2023), object detection (Jawaharlalnehru et al., 2022; Magalhães et al., 2023), natural language processing (Haq et al., 2022), and speech recognition (Kumar et al., 2023). In recent years, DL has become increasingly popular because, unlike traditional machine learning methods, it has the ability to learn more complex patterns in large datasets. DL has been used to solve various problems in different fields such as climate change prediction and environmental analysis (Haq et al., 2021a; Haq, 2022b; Haq, 2022). Therefore, DL is a powerful tool that has the potential to address many of the world’s most pressing problems. 1. CNN based automated weed detection system using uav imagery; 2. Fruit image classification model based on MobileNetV2 with deep transfer learning technique; 3. Deep learning based supervised image classification using uav images for forest areas classification; 4. Target object detection from unmanned aerial vehicle (uav) images based on improved yolo algorithm; 5. Benchmarking edge computing devices for grape bunches and trunks detection using accelerated object detection single shot multibox deep learning models; 6. Insider threat detection based on nlp word embedding and machine learning; 7. Multilayer neural network based speech emotion recognition for smart assistance; 8. Smotednn: a novel model for air pollution forecasting and aqi classification; 9. Cdlstm: a novel model for climate change forecasting; 10. Deep learning based modeling of groundwater storage change.

Currently, alien invasive plants have caused extensive and serious harm to crops (Monteiro and Santos, 2022). Invasive plants compete with crops for resources such as nutrients, water, and sunlight in the soil, affecting the growth environment of crops and reducing their growth rate and yield. Ambrosia trifida, commonly known as a foreign invasive plant in China, is considered one of the weeds causing the greatest economic losses to wheat and other annual crops (Kong et al., 2007). Furthermore, due to its allergenic pollen and presence in urban areas, Ambrosia trifida has been identified as a public health issue (Hovick et al., 2018). Therefore, effectively identifying and managing Ambrosia trifida, taking reasonable prevention and control measures, is crucial to ensuring crop yield and quality, improving the efficiency of farmland, and ensuring the normal lives of residents.

Monitoring harmful plants using remote sensing imagery has been a hot research topic in recent years. In comparison to the cost and limitations of ground-based observation and satellite remote sensing (Radoglou-Grammatikis et al., 2020), unmanned aerial vehicles (UAVs) have garnered attention due to their low-altitude flying capability and efficient operations (Tsouros et al., 2019). UAV plant image detection technology involves using high-resolution cameras mounted on UAVs to capture plant image data for analysis and processing, enabling rapid, automated identification and classification of plants (Betti, 2022). Equipped with high-performance cameras, UAVs can accurately and swiftly acquire large-scale, high-resolution field images and achieve high-precision identification of plants. Additionally, researchers can freely control UAV flights based on specific needs and field conditions (Wang Z. et al., 2022), which significantly enhances the detecting efficiency.

The existing plant image detection methods for UAVs can be mainly classified into two categories: those based on specific features and those based on abstract features (Mittal et al., 2020). The methods based on specific features primarily utilize manually designed features such as SIFT (Scale Invariant Feature Transform), HOG (Histogram of Oriented Gradient), and SURF (Speeded-Up Robust Features) to represent the targets. These features provide local information and texture characteristics of the targets, which are then used for target classification and position regression using traditional machine learning algorithms. This method is somewhat limited by the accuracy of the manually designed features. If these features cannot adequately express the abstract features of weeds, they may not adapt well to scenes with dense weed distribution and partial occlusion, thus limiting their performance (Dong et al., 2021). With the improvement of computer performance, computer vision based on Convolutional Neural Networks (CNN) has made great progress (Bhatt et al., 2021). These methods mainly use deep neural networks (DNN) to automatically learn abstract features to represent the targets. By constructing convolutional neural networks or other types of neural networks, they can directly learn the abstract representation of the targets from the original images and perform target classification and position regression (Sarker, 2021). These abstract features are extracted by convolutional neural networks without human intervention, and therefore, these methods usually have higher detection accuracy, and are commonly used for implementing field weed segmentation and detection.

One-Stage and Two-Stage detection algorithms are the two major categories of detection methods based on abstract features. The Two-Stage detection algorithms mainly include SPP-Net (He et al., 2015), Fast R-CNN (Girshick, 2015), and Faster R-CNN (Ren et al., 2015). The Two-Stage detection algorithm is divided into two stages: in the first stage, a series of candidate regions are generated through the Region Proposal Network (RPN), and in the second stage, classification and regression operations are performed on all the candidate regions to obtain the detection results (Wu et al., 2020). The One-Stage detection algorithm, as a regression-based object detection method, can directly predict the target category and location from the input image, usually requiring only one forward pass to complete the detection process. The current mainstream One-Stage detection algorithms include SSD (Liu et al., 2016) and the YOLO family, including YOLO (Redmon et al., 2016), YOLO9000 (Redmon and Farhadi, 2017), YOLOv3 (Redmon and Farhadi, 2018), YOLOv4 (Bochkovskiy et al., 2020), YOLOv5 (Ultralytics,), YOLOv7 (Wang et al., 2023), DC-YOLOv8 (Lou et al., 2023), YOLO-NAS (Terven et al., 2023) and RT-DETR (Lv et al., 2023). YOLOv5 detection algorithm, as a type of One-Stage detection algorithm, has a faster detection speed compared to Two-Stage detection algorithms. However, due to the small size, high density, and overlapping characteristics of Ambrosia trifida images captured by drones, applying the YOLOv5 detection algorithm to detect Ambrosia trifida can easily lead to false positives and missed detections, this results in low accuracy in detecting Ambrosia trifida in drone images.

In order to increase the detection accuracy of Ambrosia trifida in UAV images, this paper introduces a novel UAV image detection algorithm called YOLOv5-KE based on YOLOv5. YOLOv5-KE enhances the original YOLOv5 network by adding a micro-scale detection layer, adjusting the Anchor Box hierarchical detection settings based on k-Means, improving the loss function of CIoU, and implementing a detection box fusion mechanism based on confidence weight to merge detection boxes in multi-resolution images. This aims to improve the feature extraction capability and detection accuracy of Ambrosia trifida in UAV images under conditions of small targets and partial occlusion. Comparative validation experiments conducted on a self-built dataset of Ambrosia trifida demonstrate that YOLOv5-KE can accurately detect multi-scale Ambrosia trifida in complex field environments and shows potential in detecting weeds with high-density and overlapping leaves. It provids technical references for similar plant detecting conditions.

## 2 Materials and methods

### 2.1 YOLOv5 network structure

**Figure 1:**
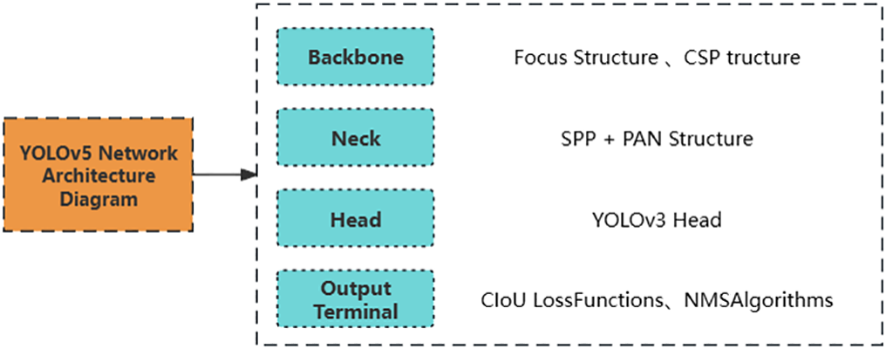
Network architecture diagram of YOLOv5.

YOLOv5 is a neural network released by Glenn Jocher in 2020, and its network structure consists of four parts: Backbone network, Neck network, Head network, and the output end, as shown in **Figure 1** (Wu et al., 2021). The Backbone network of YOLOv5 mainly consists of the Focus structure and the CSP structure. The Focus structure is a convolutional neural network layer used for feature extraction, compressing and combining information from the input feature map to extract higher-level feature representations. The CSP (Cross Stage Partial) structure effectively reduces network parameters and computational complexity while improving feature extraction efficiency. The intermediate Neck network of YOLOv5 is primarily used to enhance the model’s feature expression capability and receptive field, further improving the model’s detection performance (Kattenborn et al., 2021). YOLOv5 uses two different Neck network structures: SPP (Spatial Pyramid Pooling) and PAN (Path Aggregation Network). The SPP structure is a pyramid pooling structure that can pool feature maps of different sizes to enhance the model’s perception of targets at different scales. PAN is a feature pyramid network structure for object detection designed to enhance the model’s perception of targets at different scales through multi-level feature fusion. The SPP structure enhances the model’s perception and scale invariance, while the PAN structure enhances the fusion ability of multi-scale features. The SPP and PAN structures can be used in combination to improve the model’s detection performance. The Head network of YOLOv5 is the same as the Head network of YOLOv3. The bounding box loss function used at the output end of YOLOv5 is the CIoU Loss function, which further introduces the concept of corner distance based on GIoU, effectively alleviating the impact of rotation and tilt on target detection performance, thereby improving the model’s performance (Ding et al., 2022).

### 2.2 YOLOv5-KE network structure

**Figure 2:**
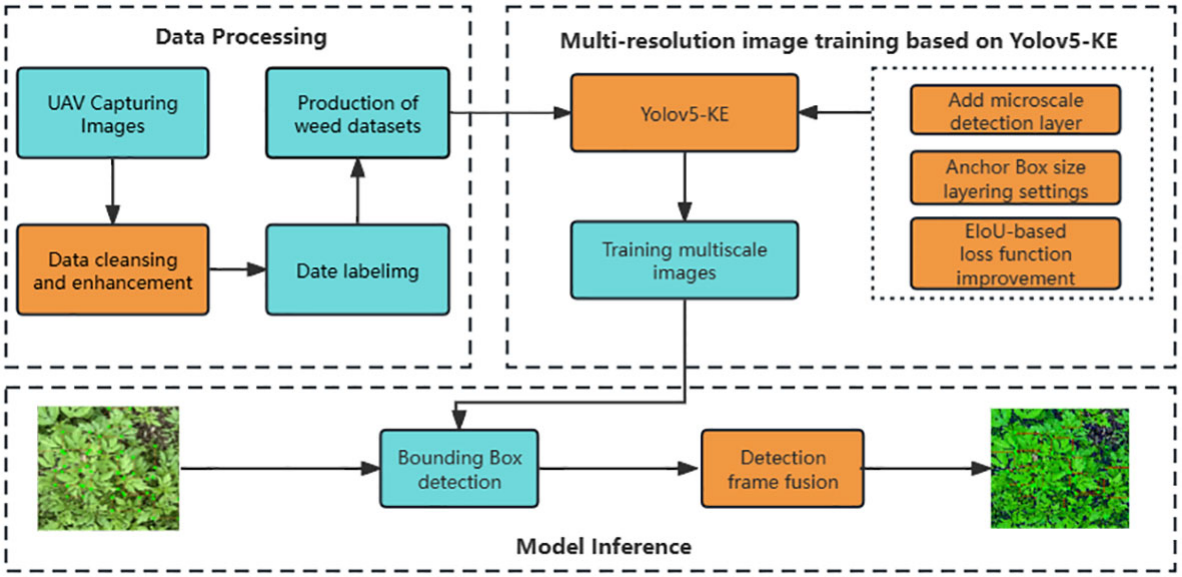
Schematic diagram of YOLOv5-KE application.

The YOLOv5-KE algorithm proposed in this paper is based on the YOLOv5 algorithm, with the addition of a micro-scale detection layer, adjustments to the hierarchical detection settings of Anchor Boxes based on k-Means, improvements to the loss function of CIoU, and the reselecting Weighted Box Fusion (WBF) algorithm as the method for calculating the fusion of detection boxes. The network structure is illustrated in **Figure 2**.

#### 2.2.1 Micro-scale detection layer

**Figure 3:**
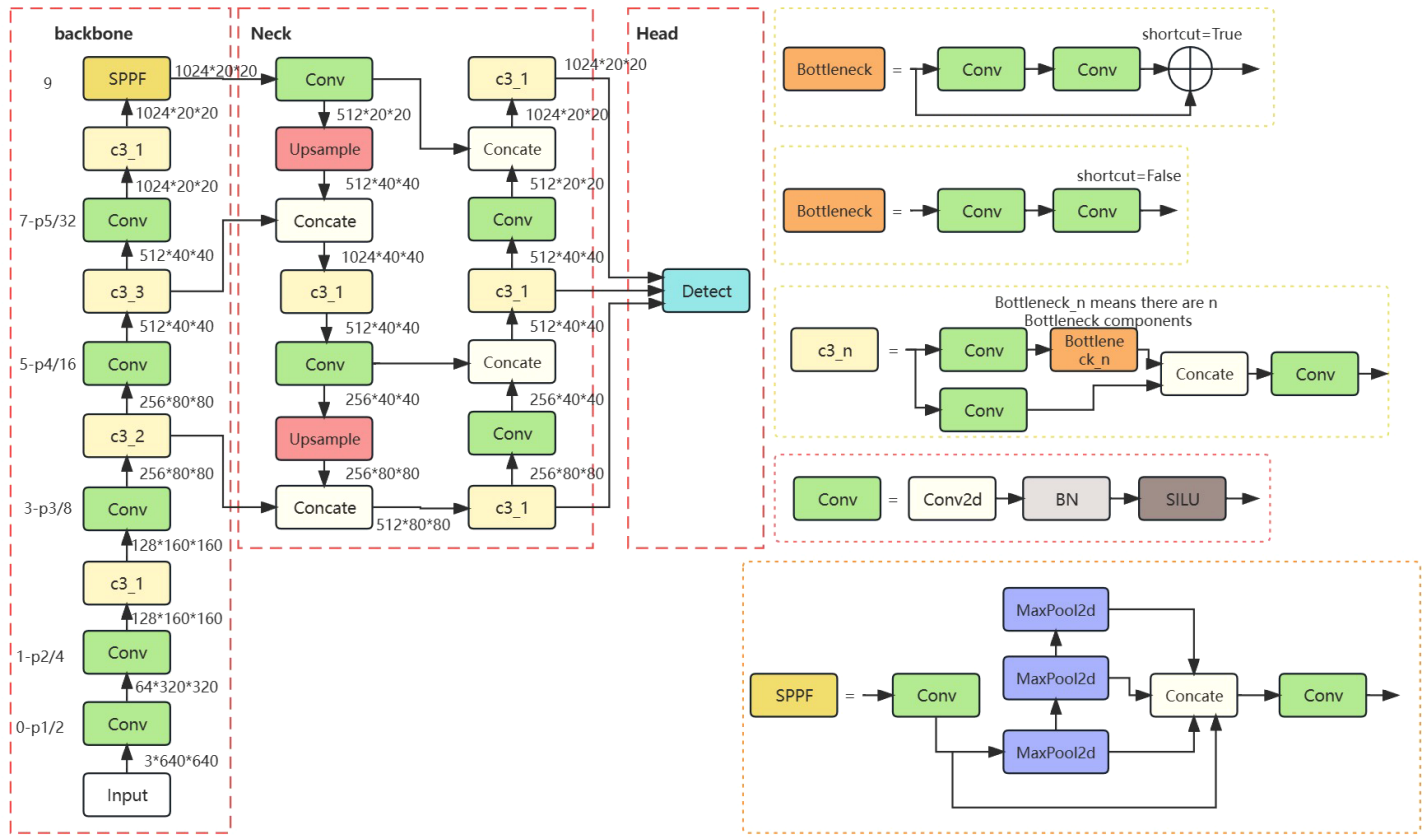
YOLOv5 original network structure.

**Figure 4:**
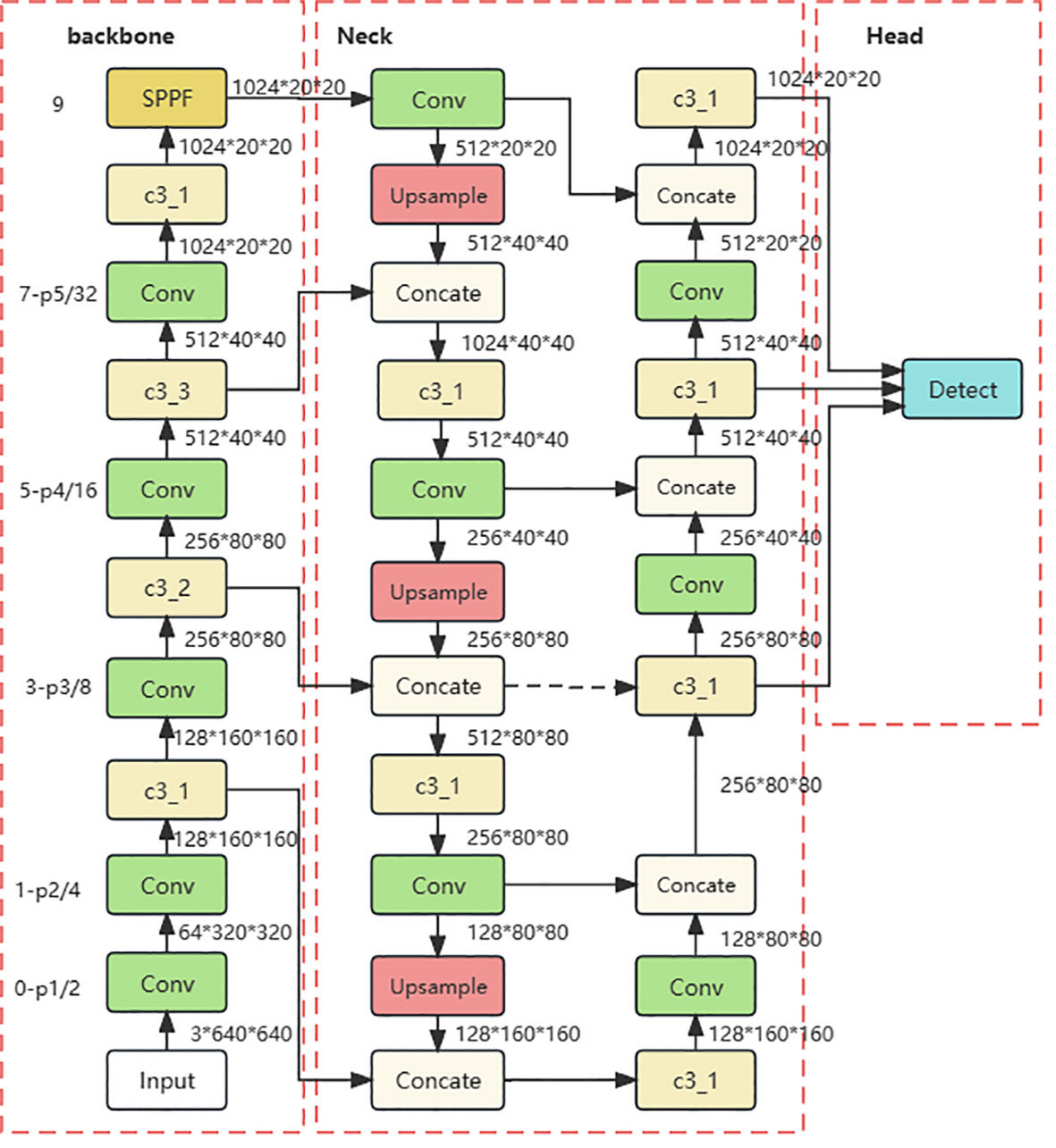
YOLOv5 network structure with the addition of a microscale detection layer.

The head network of YOLOv5 has three scale detection layers, enabling detection at three different scales: 80x80 (small targets), 40x40 (medium targets), and 20x20 (large targets). Each layer is equipped with anchors of varying sizes. On the 8x downsampled feature map (80x80), three types of anchors are placed at each feature point, with respective widths and heights of (10, 13), (16, 30), and (33, 23). Similarly, on the 16x downsampled feature map (40x40), three anchors are placed with widths and heights of (30, 61), (62, 45), and (59, 119). On the 32x downsampled feature map (20x20), three anchors with widths and heights of (116, 90), (156, 198), and (373, 326) are placed. When the training dataset contains diverse and complex objects with significant variations in size, the use of multi-scale and multi-anchor detection in the model effectively enhances detection accuracy, the detailed network of YOLOv5 is illustrated in **Figure 3**. However, the small-scale detection layer of the YOLOv5 network is not well-suited for recognizing Ambrosia trifida in drone images, as these plants are small in size and densely distributed. Therefore, the YOLOv5-KE detection algorithm proposed in this paper introduces a new micro-scale detection layer into the YOLOv5 model, as depicted in **Figure 4**. This detection layer upsamples the 80x80 feature map from the NECK to 160x160, adds it to the 160x160 feature map from the backbone network, and then downsamples it back to 80x80. By extracting spatial features from the lower layers and fusing them with deep semantic features to generate the feature map, the YOLOv5-KE detection network structure becomes more comprehensive and detailed, suitable for detecting the tiny Ambrosia trifida in drone images.

#### 2.2.2 k-Means based anchor box hierarchical detection

Anchor Box is a concept proposed by Ross Girshick et al. in 2015 in the Faster RCNN network to detect multiple objects within grid cells (Jiao et al., 2020). The YOLOv5 detection network uses automatic Anchor Boxes to well match the detected objects (Zhong et al., 2020). Anchor Boxes heavily rely on pre-learning from the dataset. In previous studies, Anchor Boxes were automatically learned from the entire dataset and performed well on datasets with relatively uniform scales. However, in this study, the size of Ambrosia trifida in drone images varies significantly, and the quantity of samples of different sizes is uneven. During detection, the YOLOv5 detection network tends to focus more on Ambrosia trifida of the same size but with greater numbers, resulting in lower recognition accuracy for Ambrosia trifida with fewer instances. For these reasons, this research sets up 4 detection layers and categorizes all Ambrosia trifida into 4 groups based on their sizes, aiming to increase the attention of the YOLOv5-KE detection network to Ambrosia trifida with fewer individual sizes. The specific approach is to categorize all Ambrosia trifida into 4 classes based on their sizes { $G_{i}$ }= $G_{i=1}^{4}$ . For all Ambrosia trifida $\;gt_{j}^{i}$ ( $x_{j}$ , $y_{j}w_{j}$ , $z_{j}$ ), j ∈{ 1,…M}, i ∈ {1,…N} in each class $\;G_{i\;}$ , the distance measure between the ground truth box and the Anchor Box can be defined as:


(1) d(gt,bbox)=1−IoU(gt,bbox)

(2) $$\;IoU\left(gt,bbox\right)=\frac{area\left(gt\cap bbox\right)}{area\left(gt\cup bbox\right)}\;$$

In the equation: gt represents the true box position of Ambrosia trifida, and bbox represents the Anchor Box. The larger the IoU value between gt and bbox , the smaller the distance measurement, the more accurately that Anchor Box describes the position of Ambrosia trifida. Each class $G_{i}$ introduces a different Anchor box, enabling the detection of Ambrosia trifida of different sizes in unmanned aerial vehicle images. The clustering process is shown in Algorithm 1.

Algorithm 1. Program to set the size of the anchors box
**Input:** Ground truth box 
$G_{i}$

**Output:** anchor boxes 
$C_{j}$

1: Randomly select K points from the data set as the clustering center point 
$C_{j}=\left\{C_{1},C_{2},\ldots \ldots ,C_{K}\right\}$

2: **Repeat**
3:   Steps.
4:      Calculated distance between 
$C_{j}$
 and 
$\;G_{i}$
 from Equations (1),(2)
5:      Recalculate the center of clustering from Equation (3),(4)
6: **Until the** center of the cluster converges

(3) $${\text{H}}_{\text{i}}^{\text{o}+1}=\frac{1}{{\text{N}}_{\text{i}}}\sum {\text{H}}_{\text{i}}^{\text{o}}$$

(4) $${\text{Z}}_{\text{i}}^{\text{o}+1}=\frac{1}{{\text{N}}_{\text{i}}}\sum {\text{Z}}_{\text{i}}^{\text{o}}$$

which ${\text{H}}_{\text{i}}^{\text{o}+1}$ and ${\text{Z}}_{\text{i}}^{\text{o}+1}$ are the new clustering centers for computing the new distance metric.

#### 2.2.3 Improvement of loss function algorithm

The CIoU loss function used in the YOLOv5 neural network is defined as Equation (5) in reference (Liu and Dai, 2023). This loss function, building upon the DIoU loss function, incorporates a measure of the aspect ratio difference between the predicted box and the ground truth box, which can potentially accelerate the regression speed of the predicted box and make it more consistent with the real box. However, it still suffers from significant issues: the parameter ν in the CIoU loss function reflects the difference in aspect ratio rather than the actual differences in width and height compared to their confidence levels. As a result, this may hinder the model’s optimization and reduce the convergence speed (Du et al., 2021).

To address this issue, this study proposes the EIoU loss function, integrating Focal-enhanced high-quality anchor boxes, known as the Focal-EIoU loss, to improve the convergence speed and localization accuracy of the loss function. The penalty term in EIOU is based on the penalty term in CIOU, where the influence factor of the aspect ratio is separately calculated for the length and width of the target box and the anchor box (Mohammed et al., 2022). This loss function comprises three components: overlap loss, center distance loss, and width-height loss. The first two components follow the methods in CIOU, but the width-height loss directly minimizes the difference in width and height between the target box and the anchor box, leading to faster convergence speed. Its definition is shown in Equation (6). To focus the EIoU loss on high-quality examples, the value of IOU is used to reweight the EIoU loss, resulting in the Focal-EIOU loss function as follows (Equation 7):


(5) $${\text{L}}_{\text{CIoU}}=1-\text{IOU}+\frac{{\text{ρ}}^{2}\left(\text{b},{\text{ b}}^{\text{gt}}\right)}{{\text{c}}^{2}}+\text{αν}$$

(6) $$\begin{array}{l} \;{ℒ}_{\text{EIoU}}={ℒ}_{\text{IoU}}+{ℒ}_{\text{dis}}+{ℒ}_{\text{asp}}\\ =1-\text{IoU}+\frac{{\text{ρ}}^{2}(\text{b},{\text{ b}}^{\text{gt}})}{{\left({\text{w}}^{\text{c}}\right)}^{2}+{\left({\text{h}}^{\text{c}}\right)}^{2}}+\frac{{\text{ρ}}^{2}(w,w^{\text{gt}})}{{\left({\text{w}}^{\text{c}}\right)}^{2}}+\frac{{\text{ρ}}^{2}(h,k^{\text{gt}})}{{\left({\text{h}}^{\text{c}}\right)}^{2}}\; \end{array}$$

(7) $${\text{L}}_{\text{Focal}-\text{EIoU}}={\text{IOU}}^{\text{γ}}{\text{L}}_{\text{EIoU}}\;$$

Where IOU=|A∩B|/|A∪B| , the γ is A parameter that controls the degree of outlier suppression. In Formula 5, ρ represents the normalized Euclidean distance between the center points of the predicted and ground truth bounding boxes. c represents the diagonal length of the smallest enclosing box containing both the predicted and ground truth bounding boxes. a is a balancing parameter used to adjust the importance of the regularization term v. v is a regularization term used to penalize the aspect ratio discrepancy between the predicted and ground truth bounding boxes.

#### 2.2.4 Improvement of detection box fusion algorithm

The fusion of detection boxes based on confidence weighting is a common step in object detection algorithms (Shen et al., 2022). In object detection tasks, multiple different models or algorithms are often used to generate candidate detection boxes, which may exhibit some degree of overlap or redundancy. To improve the accuracy and stability of the detection results, it is necessary to merge these candidate boxes.

The common fusion strategy in YOLOv5 is the Non-Maximum Suppression (NMS) method (Solovyev et al., 2021), which selects the most representative and accurate detection results by comparing the degree of overlap between the candidate boxes. However, the NMS algorithm typically assumes that the targets are relatively simple geometric shapes, such as rectangles or circles, and that the sizes of the targets should be relatively consistent with each other (Hosang et al., 2017). In the case of identifying Ambrosia trifida, the leaves of Ambrosia trifida may be very crowded, and the size of leaves may be significant variations, which cause the NMS algorithm to incorrectly merge candidate detection boxes. Additionally, if there is partial overlap or occlusion among Ambrosia trifida in the image, the NMS algorithm may not able to accurately determine the boundaries and positions of the targets, leading to merging errors or missed detections. This problem may be exacerbated when Ambrosia trifida is densely distributed (Solovyev et al., 2021).

**Figure 5:**
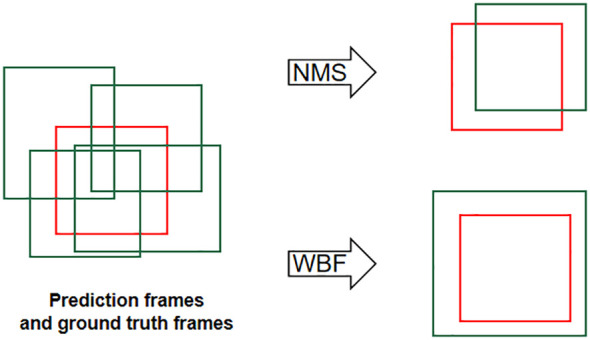
Schematic diagram of non-maximum suppression (NMS) and weighted box fusion (WBF).

**Figure 6:**
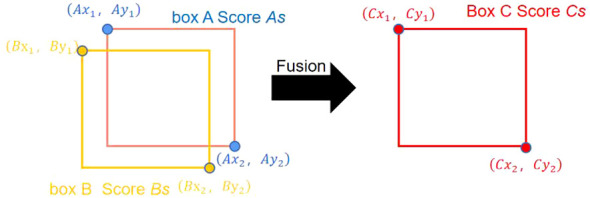
WBF algorithm fusion process.


For these reasons, this study adopts the Weighted Box Fusion (WBF) algorithm as the fusion algorithm for YOLOv5-KE. The WBF algorithm enhances the performance of object detection by merging detection results from multiple scales (Zhang et al., 2023). The fusion weight calculation in WBF is described by Equations (8–10), and the illustration of detection box generation and the fusion process are shown in **Figures 5**, **6**. In **Figure 5**, the green boxes represent the predicted boxes, while the red boxes represent the ground truth boxes. In **Figure 6**, the scores of the boxes are used as weights, and the coordinates of the two boxes are merged to obtain a new box. Therefore, boxes with higher scores have greater weights, and their contributions are more significant in the process of generating the new box. The shape and position of the new box are biased towards the box with a higher weight (Zhai and Zhuang, 2020).

(8) $${\text{ C}}_{{\text{x}}_{1}}=\frac{{\text{A}}_{{\text{x}}_{1}}\times {\text{A}}_{\text{s}}+{\text{B}}_{{\text{x}}_{1}}\times {\text{B}}_{\text{s}}}{{\text{A}}_{\text{s}}+{\text{B}}_{\text{s}}}{\text{ C}}_{{\text{y}}_{1}}=\frac{{\text{A}}_{{\text{y}}_{1}}\times {\text{A}}_{\text{s}}+{\text{B}}_{{\text{y}}_{1}}\times {\text{B}}_{\text{s}}}{{\text{A}}_{\text{s}}+{\text{B}}_{\text{s}}}\;$$

(9) $${\text{C}}_{{\text{x}}_{2}}=\frac{{\text{A}}_{{\text{x}}_{2}}\times {\text{A}}_{\text{s}}+{\text{B}}_{{\text{x}}_{2}}\times {\text{B}}_{\text{s}}}{{\text{A}}_{\text{s}}+{\text{B}}_{\text{s}}}{\text{ C}}_{{\text{y}}_{2}}=\frac{{\text{A}}_{{\text{y}}_{2}}\times {\text{A}}_{\text{s}}+{\text{B}}_{{\text{y}}_{2}}\times {\text{B}}_{\text{s}}}{{\text{A}}_{\text{s}}+{\text{B}}_{\text{s}}}\;$$

(10) $${\text{C}}_{\text{s}}=\frac{{\text{A}}_{\text{s}}+{\text{B}}_{\text{s}}}{2}\;$$

### 2.3 Image data acquisition and processing

#### 2.3.1 Data acquisition

**Figure 7:**
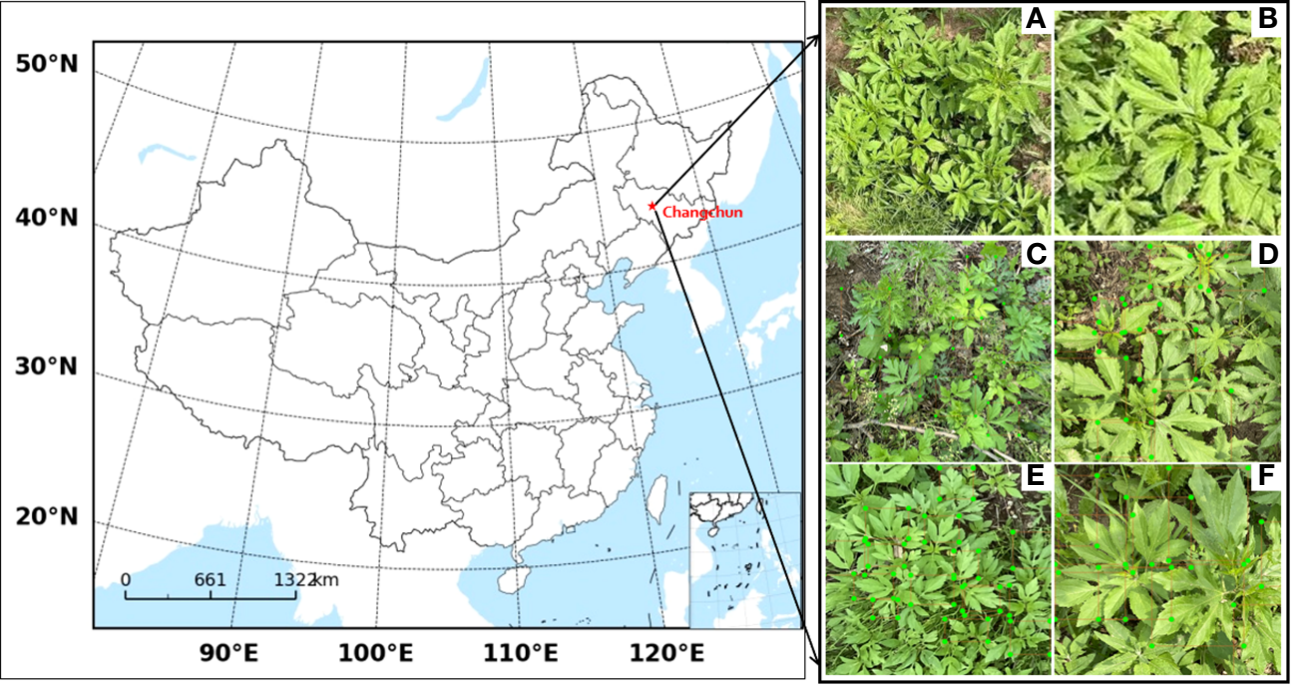
Images of Ambrosia trifida taken at data collection sites and by drones **(A)** Cropped image **(B)** Laplace transformed image **(C-F)** Artificially labeled image.

The images of Ambrosia trifida were collected in seven different areas in Changchun, Jilin Province, China at three different periods, as shown in **Table 1**; **Figure 7**. The image collection was conducted using a DJI Mavic 3 unmanned aerial vehicle equipped with a Hasselblad 4/3 CMOS camera, capturing images at heights of 5m, 10m, and 15m.

**Table 1:** Specific information of ambrosia trifida photography.

Date	Location	Growth Stage
June 2023	Changchun, Nanguan District, Jilin Agricultural University Back Mountain Changchun, Jingyue Tan National Forest Park Changchun, Erdao District, Changqing Village Farmland	Seedling Stage
August 2023	Changchun, Nong’an County, Shengli Village Farmland Changchun, Jingyue Tan National Forest Park Changchun, Lvyuan District, Xixin Village Farmland	Flowering Stage
September 2023	Changchun, Jiutai District, Xinhe Town Farmland Changchun, Kuancheng District, Qianlou Village Farmland Changchun, Jingyue Tan National Forest Park	Fruiting Stage

To reduce data processing time, highlight the characteristics of Ambrosia trifida, and avoid loss of image information, the original images with dimensions of 5280×3956 pixels were cropped to 640×640 pixels (**Figure 7A**). Additionally, due to the potential instability during drone flights, some blurred images were obtained. To address this, Laplacian transformation was applied to remove the blurred images (Yakovlev and Lisovychenko, 2020), resulting in clear images (**Figure 7B**). The clear images of Ambrosia trifida were individually annotated using the image annotation tool LabelImg (**Figures 7C-F**).

#### 2.3.2 Data enhancement

**Figure 8:**
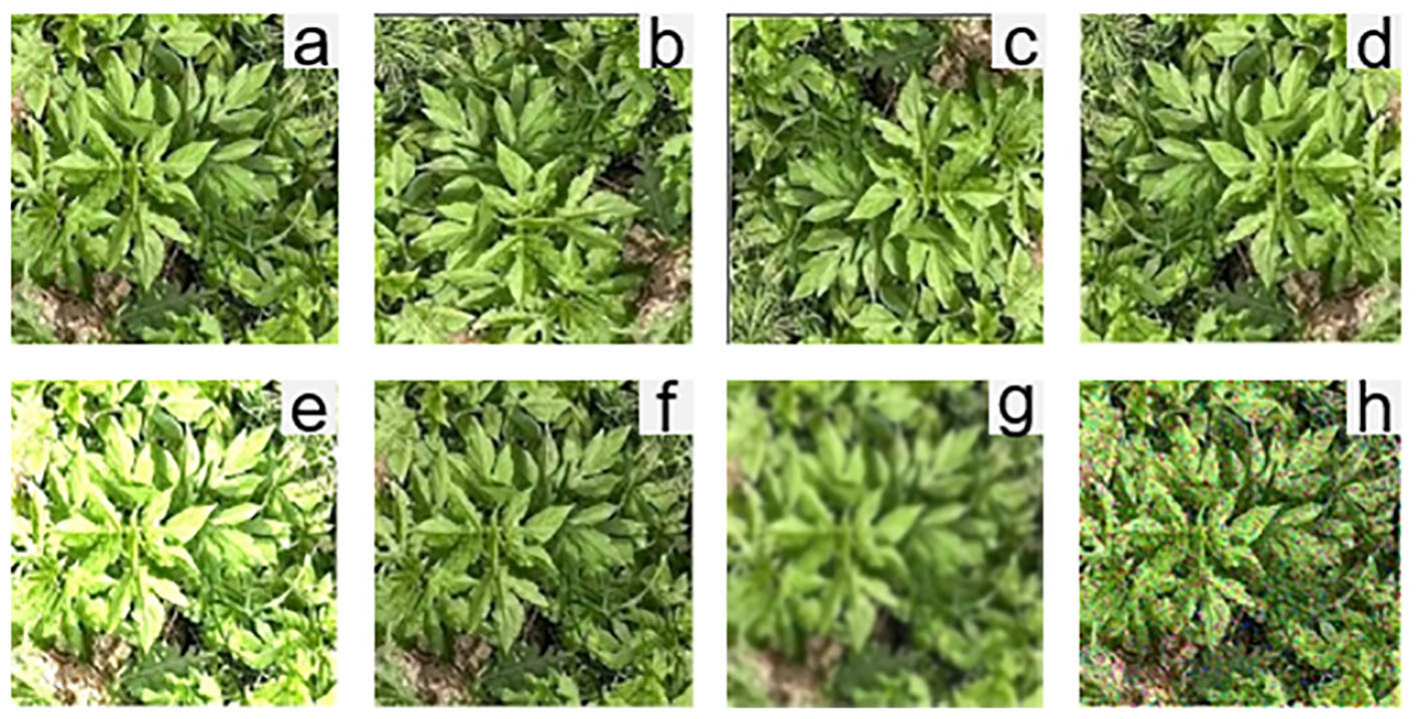
Data enhancement **(A)** original image, **(B)** rotated by 90°, **(C)** rotated by 180°, **(D)** flipped horizontally, **(E)** brightness-enhanced, **(F)** brightness-darkened. **(G)** pretzel noise, **(H)** Gaussian noise.

Data augmentation can expand the training dataset, reduce model overfitting, balance data distribution, enhance model robustness, improve generalization capabilities, and enhance detection performance. In this study, methods such as image rotation, image flipping, brightness adjustment, and adding noise were selected as data augmentation techniques, as shown in **Figure 8**. Rotating and flipping images can help the model learn a more comprehensive and diverse range of features of Ambrosia trifida, enhancing model robustness. Additionally, brightness adjustment allows the model to learn target features under different lighting conditions, improving recognition accuracy under varying illumination conditions (Mahmud et al., 2021). Noise can simulate various interferences and uncertainties in real-world situations. By adding noise, model generalization capabilities can be improved, enabling accurate detection in complex scenarios. After data augmentation, a total of 10,000 images were obtained and divided into training, validation, and test datasets in a 7:2:1 ratio.

#### 2.3.3 Data resampling

**Figure 9:**
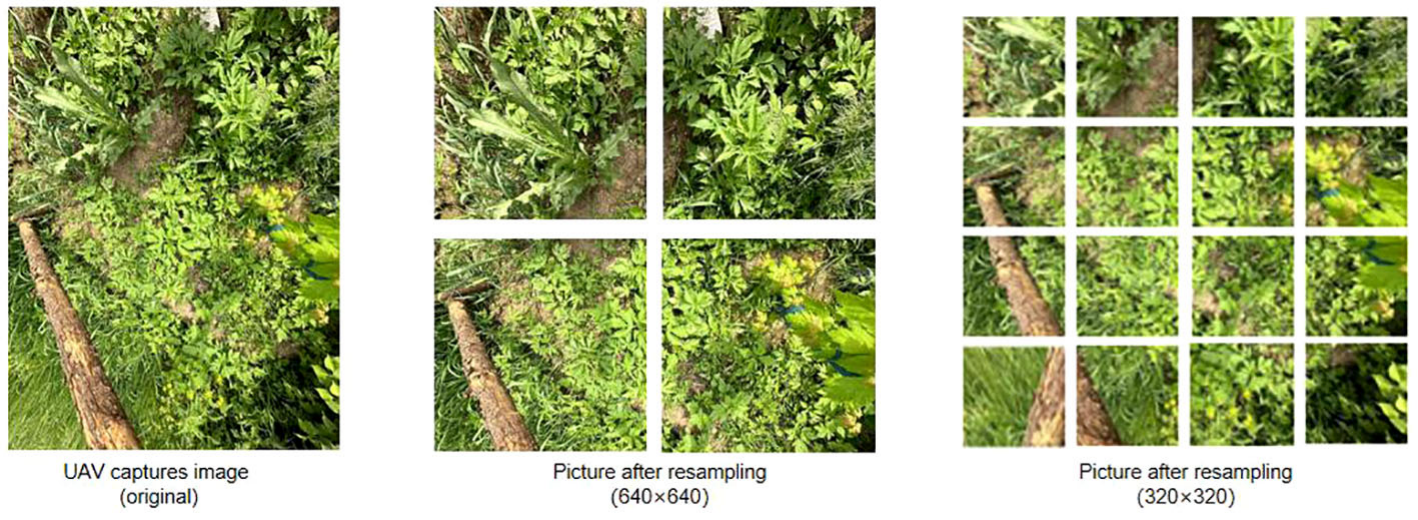
Resampling of Ambrosia trifida pictures.

In general, images in the dataset are processed to have the same resolution before training to ensure consistency and comparability of the data. In contrast to previous studies that only trained on single-resolution images (Sharma et al., 2020), this study resamples the Ambrosia trifida images to train on multiple resolutions. Training on multi-resolution images provides broader coverage and leads to a more stable model. After resampling, the Ambrosia trifida images are divided into four resolutions: 100×100, 160×160, 320×320, and 640×640. as shown in **Figure 9**.

## 3 Experiments on Ambrosia trifida recognition based on YOLOv5-KE

### 3.1 Experimental process

**Figure 10:**
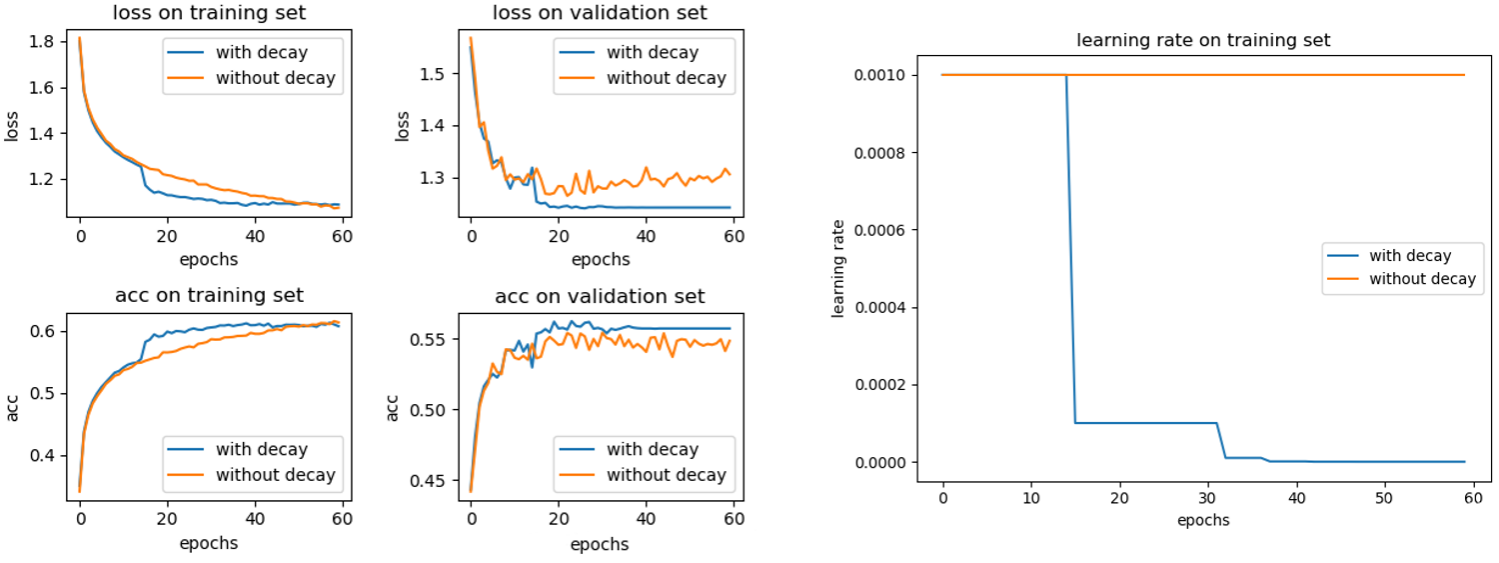
Adding a comparison between before and after learning rate decay.

To verify the higher detection accuracy of YOLOv5-KE compared to existing neural networks for Ambrosia trifida, we trained the aforementioned network models using a self-built dataset. The training devices consisted of an Intel i5-13600 processor, NVIDIA 3070Ti graphics processor (16GB memory), and 1TB RAM, with Windows 10 as the operating system. During training, we adopted the Adam (Adaptive Moment Estimation) optimizer with learning rate decay to optimize learning efficiency. By decaying the learning rate, the model was allowed to use a larger learning rate in the early stages to accelerate convergence, and then decreased the learning rate in later stages for better parameter adjustment. This approach improves the performance and stability of the model, preventing issues like overfitting or getting trapped in local optima (**Figure 10**).

For the resampled images at different resolutions, the training parameters were set as follows: momentum of 0.99, decay factor of 0.5, 300 epochs, and decay weight of 0.0001. The specific settings for training model hyperparameters are shown in **Table 2**. Using Hyperopt for automated hyperparameter tuning with the provided parameters, opt for the Tree of Parzen Estimators(TPE) algorithm to achieve the optimal hyperparameter combination.

**Table 2:** Specific settings for training model hyperparameters.

Input Resolution	Batch size	Epoch	Iterations	Momentum	learning rate	Decay Factor	Decay Weight
100 x 100	64	300	50	0.99	0.02	0.5	0.0001
160 x 160	32	300	100	0.99	0.01	0.5	0.0001
320×320	16	300	200	0.99	0.005	0.5	0.0001
640×640	8	300	400	0.99	0.0025	0.5	0.0001

To determine the most suitable drone flying height and input resolution, we trained the images captured by the drone at heights of 3m, 5m, and 10m on YOLOv5-KE, YOLOv5, YOLOv7, DC-YOLOv8, YOLO-NAS, RT-DETR, SSD, Faster RCNN, and Retina Net neural network models, using input resolutions of 100×100, 160×160, 320×320, and 640×640, respectively.

The neural networks were evaluated based on the following performance parameters: Precision (P), Recall (R), F1-score, and Average Precision (AP) (Zhou et al., 2021). Frames Per Second (FPS) was used as an indicator of detection speed. Precision (P), Recall (R), and F1-score are defined by Equations (11–13) respectively.


(11) $$\text{P}=\frac{\text{TP}}{\text{FP}+\text{TP}}$$

(12) $$\text{R}=\frac{\text{TP}}{\text{FP}+\text{TP}}\;$$

(13) $$\text{F}1=\frac{2\text{PR}}{\text{P}+\text{R}}=\frac{2\text{TP}}{2\text{TP}+\text{FN}+\text{FP}}\;$$

Among them, TP (True Positive) represents the cases where both the algorithm in this study and manual annotation successfully detected Ambrosia trifida in the drone images. TN (True Negative) represents the cases where the algorithm failed to detect Ambrosia trifida, but it was detected by manual annotation. FP (False Positive) represents the cases where the algorithm detected Ambrosia trifida, but manual annotation did not. FN (False Negative) represents the cases where neither the algorithm nor manual annotation detected Ambrosia trifida in the drone images. During the detection process, if the Intersection over Union (IoU) between the predicted bounding box and the true bounding box of Ambrosia trifida is greater than 0.5, the predicted bounding box is labeled as TP. Otherwise, it is labeled as FP. If there is no overlap between the true bounding box and any predicted bounding box, it is labeled as FN. TN is not needed in this classification process because the final detection results are fixed. TP and FP represent the correctly and incorrectly detected instances of Ambrosia trifida respectively, while FN represents the instances that were not detected. TN does not have practical significance.

Precision and recall are conflicting metrics. Generally, higher precision corresponds to lower recall, and vice versa. In this study, the identification of Ambrosia trifida has only one category, i.e., m=1, and mAP is equal to AP. Therefore, the average precision (AP) is used to represent the detection accuracy, as shown in Equation (14).


(14) $$\text{AP}=\int_{0}^{1}\text{P}\left(\text{R}\right)\text{d}\left(\text{R}\right)\;$$

AP is calculated based on the precision-recall curve and its value ranges from 0 to 1, a higher AP value indicates a higher recognition accuracy of the network.

### 3.2 Experimental results

**Figure 11:**
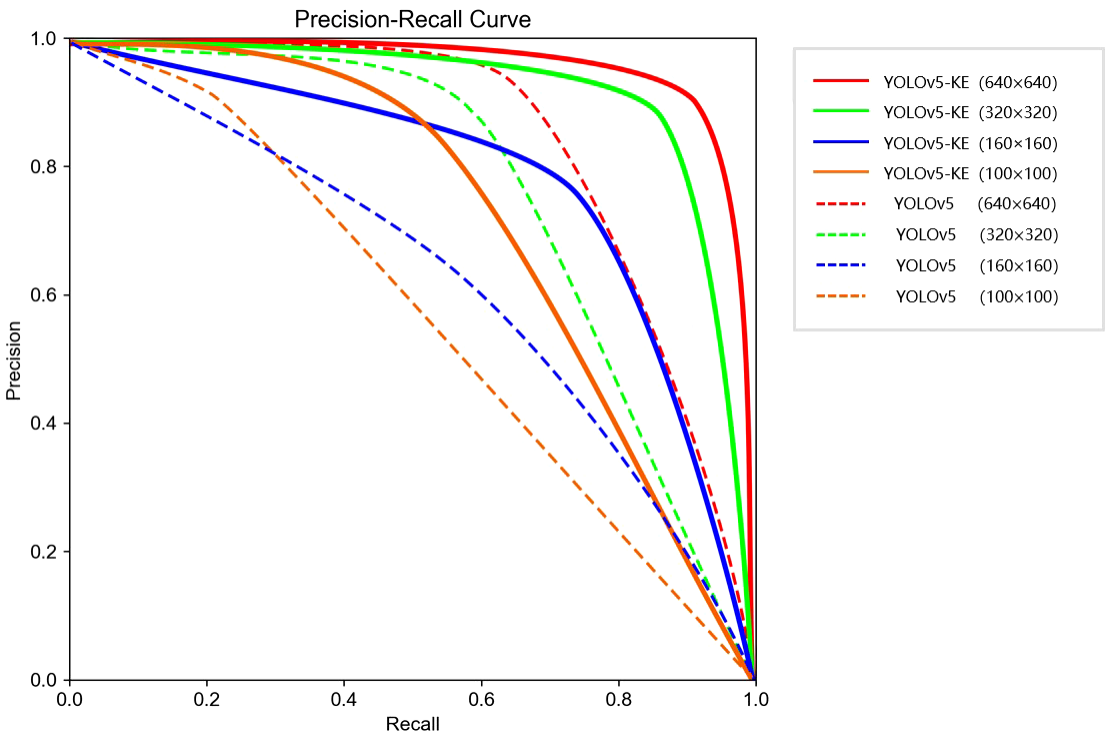
P-R curves of Ambrosia trifida images with different resolutions for YOLOv5-KE and standard YOLOv5 inputs.

The comparison experiment results are shown in **Figure 11**; **Table 3**. The experimental results indicate that there is little difference in detection speed among different models. Therefore, while maintaining a stable detection speed, we will prioritize detection accuracy as the primary evaluation metric. The results indicate that compared with the existing mainstream neural networks, YOLOv5-KE demonstrates the highest detection accuracy for Ambrosia trifida, with a precision of 93.9%. Following this, YOLOv5 achieves a precision of 78.7%, while YOLOv7 follows with a precision of 74.9%. The detection accuracy of YOLOv5-KE is 15.2% higher than the standard YOLOv5, 16.8% higher than the DC-YOLOv8 model, 15.8% higher than the YOLO-NAS model, 17.5% higher than the RT-DETR model, 19% higher than the YOLOv7 model, 38.6% higher than the SSD model, 44% higher than the Retina Net model, and the highest increase compared to Faster RCNN, reaching 59.8%.

**Table 3:** Results of comparison experiment of recognition accuracy between YOLOv5-KE and other neural network models.

Neural network	AP value (%)	Difference in AP from YOLOv5-KE (%)	FPS
YOLOv5-KE	93.9	0	30
YOLOv5	78.7	15.2	30
DC-YOLOv8	77.1	16.8	30
YOLO-NAS	78.1	15.8	30
RT-DETR	76.4	17.5	30
YOLOv7	74.9	19.0	30
SSD	55.3	38.6	35
Faster RCNN	34.1	59.8	15
Retina Net	49.9	44.0	18

The model achieves the highest detection accuracy when the input image resolution is 640×640. As the input resolution decreases, the detection accuracy of each neural network also decreases. When the resolution drops from 640×640 to 100×100, the detection accuracy of YOLOv5-KE drops from 92.6% to 62.3%. The experimental results are shown in **Table 4**.

**Table 4:** Results of detection accuracy of neural network models with different input resolutions.

Input Resolution	Neural network model	AP value (%)
100×100	YOLOv5-KE YOLOv5 DC-YOLOv8 YOLO-NAS RT-DETR YOLOv7 SSD Faster RCNN Retina Net	62.3 51.1 51.0 48.5 50.7 53.7 29.4 21.2 26.4
160×160	YOLOv5-KE YOLOv5 DC-YOLOv8 YOLO-NAS RT-DETR YOLOv7 SSD Faster RCNN Retina Net	70.0 53.2 52.0 51.7 50.7 53.9 37.8 27.8 34.6
320×320	YOLOv5-KE YOLOv5 DC-YOLOv8 YOLO-NAS RT-DETR YOLOv7 SSD Faster RCNN Retina Net	86.8 70.9 67.3 68.2 65.4 66.0 52.7 32.6 47.5
640×640	YOLOv5-KE YOLOv5 DC-YOLOv8 YOLO-NAS RT-DETR YOLOv7 SSD Faster RCNN Retina Net	93.9 78.7 77.1 78.1 76.4 74.9 55.3 34.1 49.9

The highest detection accuracy is achieved when the UAV captures images at a flying height of 5m. As the flying height increases, the accuracy gradually decreases at 10m and 15m heights. The results are shown in **Table 5**.

**Table 5:** Results of detection accuracy of drones in different models at 5m, 10m, and 15m image taking heights.

Image shooting height	neural network model	AP value (%)
3m	YOLOv5-KE YOLOv5 DC-YOLOv8 YOLO-NAS RT-DETR YOLOv7 SSD Faster RCNN Retina Net	93.9 78.7 77.9 77.0 75.3 74.8 55.3 34.1 49.6
5m	YOLOv5-KE YOLOv5 DC-YOLOv8 YOLO-NAS RT-DETR YOLOv7 SSD Faster RCNN Retina Net	81.3 66.5 66.2 65.9 64.8 65.7 43.4 25.1 40.0
10m	YOLOv5-KE YOLOv5 DC-YOLOv8 YOLO-NAS RT-DETR YOLOv7 SSD Faster RCNN Retina Net	69.1 51.8 52.0 51.1 50.7 51.4 32.2 16.4 29.3

## 4 Discussion

Comparation experimental results have shown that YOLOv5-KE and YOLOv5 outperform other neural network models such as YOLOv7, DC-YOLOv8, YOLO-NAS, RT-DETR, Faster RCNN, Single Shot MultiBox Detector (SSD), and Retina Net in the recognition accuracy of Ambrosia trifida. In fact, YOLOv5 has demonstrated excellent performance in previous weed detection tasks, achieving an average recognition rate of 90.17% for invasive weed Solanum rostratum Duna (Zaidi et al., 2022). However, in this study, the recognition accuracy of YOLOv5 for Ambrosia trifida is only 78.7%. This is because in images captured by drones, the pixels of a single Ambrosia trifida are usually 50-450 pixels, with significant size differences. In most cases, the proportion of all Ambrosia trifida weeds in the image does not exceed 40% of all field weeds. The YOLOv5 neural network has a high attention level for targets with similar sizes and large quantity, under the three detection layers, the YOLOv5 network may mistakenly detect ragweed as other beneficial plants, resulting in a significant number of missed and false detections.

Experimental results have shown that YOLOv5-KE achieves a detection accuracy of 93.9% for Ambrosia trifida when the input resolution is set to 640×640 and the UAV captures images from a height of 5 meters. This demonstrates the excellent performance of the proposed YOLOv5-KE network in recognizing Ambrosia trifida. In order to demonstrate the effectiveness of each module adjustment in YOLOv5-KE, ablation experiments were conducted using images with a resolution of 640×640 pixels, as shown in **Table 6**. It can be seen that compared to the original YOLOv5, the addition of a micro-scale detection layer improved the detection accuracy of YOLOv5-KE by 5.9%. The setting of k-Means based Anchor Box layered detection and the improvement of loss function improved the detection accuracy of YOLOv5-KE by 4.2% and 1.7%, respectively. The improvement of detection box fusion algorithm improved the detection accuracy of YOLOv5-KE by 3.4%.

**Table 6:** Results of YOLOv5-KE recognition accuracy ablation experiment results.

Microscale detection layer	Anchor Box Hierarchical Detection Based on k-Means	Improvement of EIoU-based loss function	Improvement of detection box fusion algorithm	AP value (%)
				78.7
√				84.6
√	√			88.8
√	√	√		90.5
√	√	√	√	93.9%

The symbol √ signifies the addition and utilization of corresponding improvement modules.

**Figure 12:**
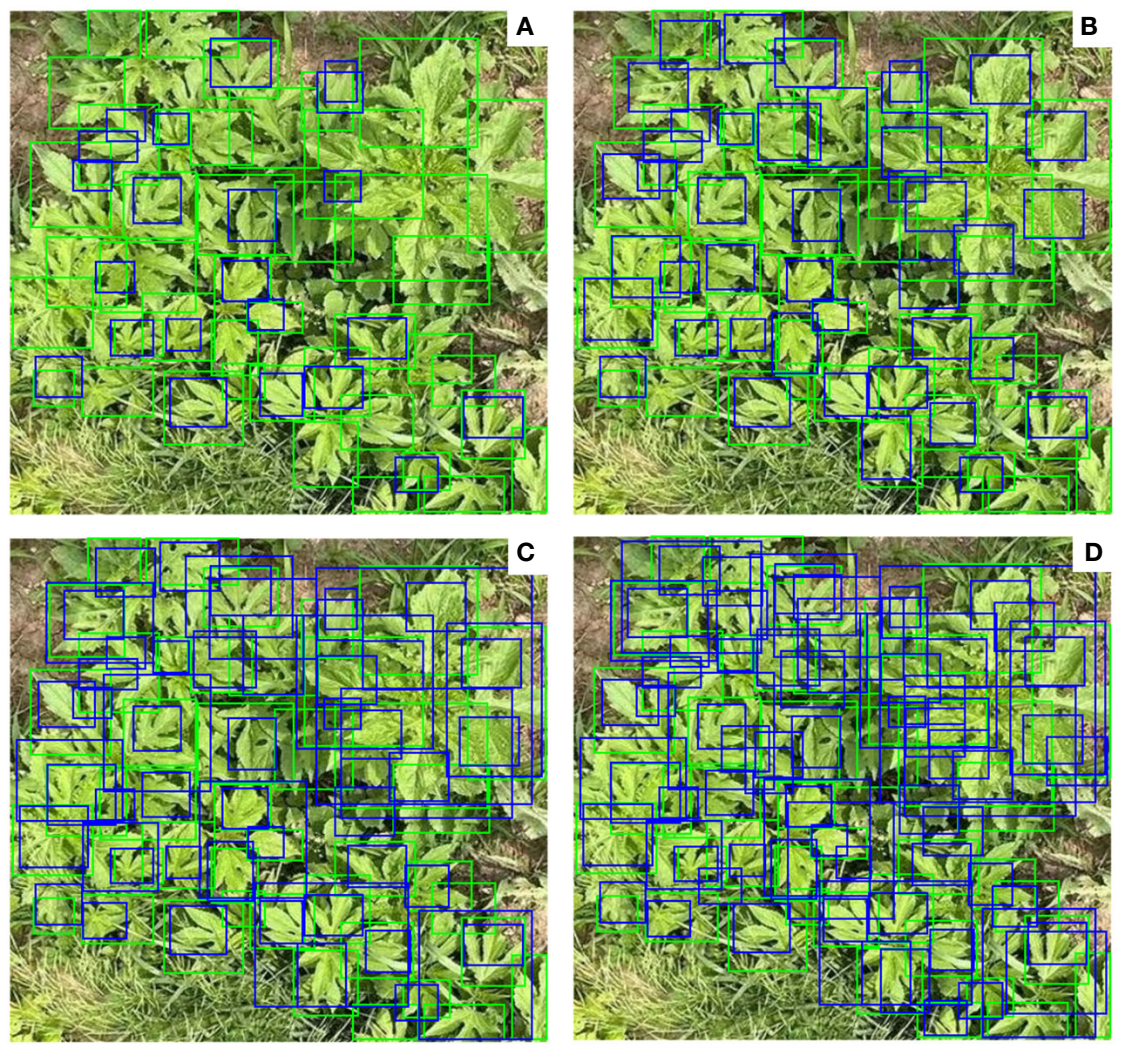
YOLOv5-KE detection anchor box (blue). Ambrosia trifida labeling position (green) **(A)** 20x20 large-target detection layer, **(B)** 40x40 medium-target labeling detection layer, **(C)** 80x80 small-target detection layer, **(D)** 160x160 microscale detection layer.

Adding a micro-scale detection layer yields the highest improvement in the recognition accuracy of Ambrosia trifida. High-quality “anchor boxes” play a crucial role in object detection models (Zaidi et al., 2022). Unfortunately, YOLOv5 cannot achieve accurate positioning of Ambrosia trifida. During the process of feature extraction, YOLOv5 reduces the resolution of the image through multiple convolution and pooling operations, i.e. downsampling. The detection layer with a higher downsampling rate corresponds to a larger receptive field and can detect larger objects. However, in this study, Ambrosia trifida in UAV images mostly appear as small targets. For small targets, detection layers with higher downsampling rates may result in inaccurate detections. The positive anchor boxes in the micro-scale detection layer are closer to the ground truth positions of Ambrosia trifida, thus contributing the most to the improved detection accuracy of YOLOv5-KE. As shown in **Figure 12**, the blue boxes represent YOLOv5-KE detection anchor boxes, while the green boxes represent the annotated positions of Ambrosia trifida. With only large target detection layers, the detection performance for Ambrosia trifida is poor, capturing only a portion of the targets. By adding medium and small target detection layers, the number of detected Ambrosia trifida increases but still misses many instances. After incorporating the micro-scale detection layer, YOLOv5-KE significantly enhances its ability to capture the feature information of small target Ambrosia trifida in the image, resulting in a substantial increase in the number of detected instances. This improvement better adapts to the scenario of high-density small-sized Ambrosia trifida targets in UAV images, leading to a noticeable enhancement in the detection performance.

**Figure 13:**
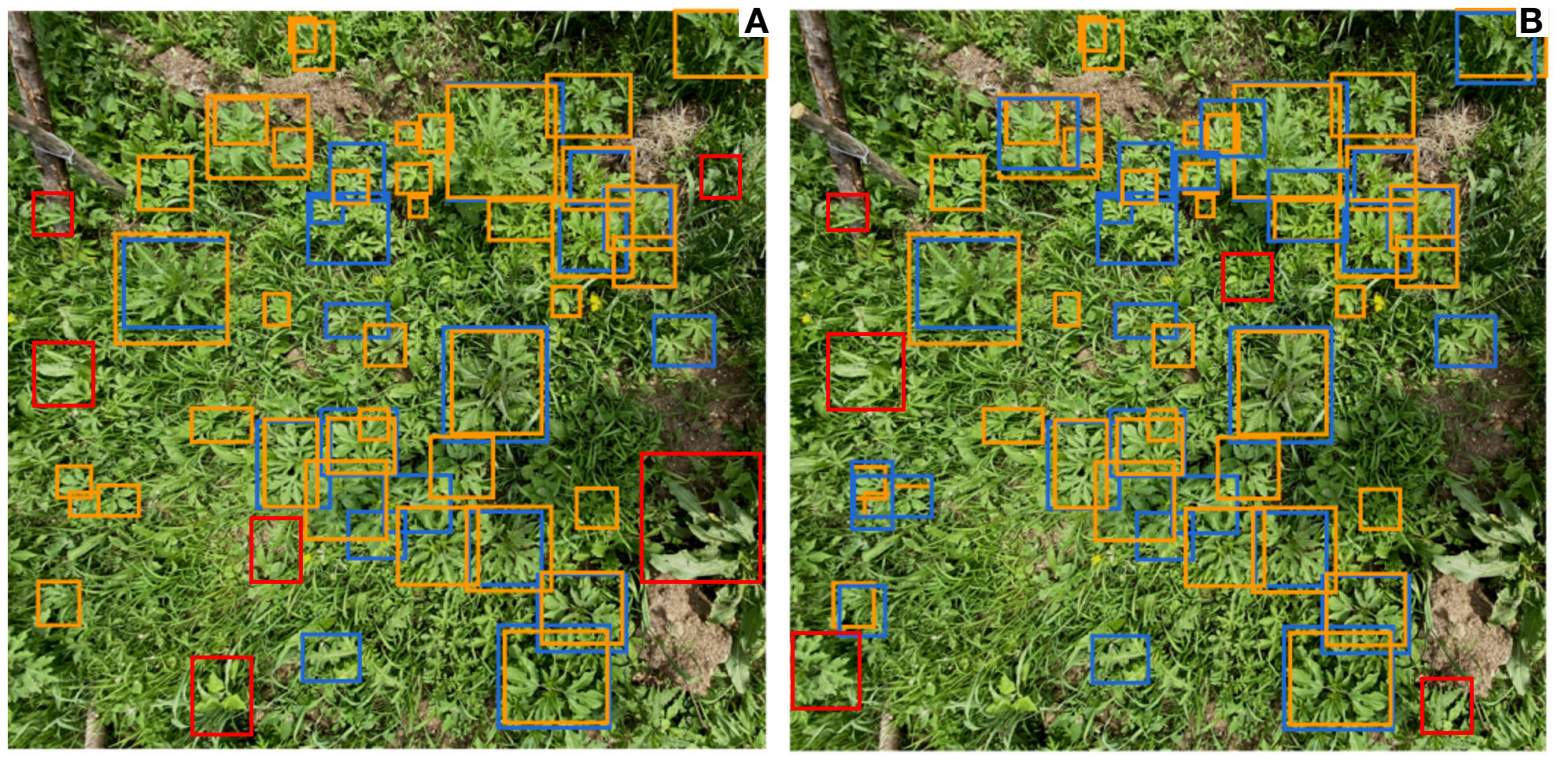
Detection results using standard Anchor Box and k-means Anchor Box **(A)** Standard Anchor Box detection results; **(B)** k-means based Anchor Box hierarchical detection results.

In the task of Ambrosia trifida detection, the setting of Anchor Boxes is a crucial issue (Gao et al., 2019). Default anchor box configurations may not yield optimal results when there are significant variations in the size of targets within the scene. Designing anchor boxes with multiple sizes and shapes based on different datasets can allow the Anchor Boxes to better focus on the characteristics of objects in the image, thus improving the accuracy of detection. The YOLOv5-KE model utilizes k-Means-based Anchor Box stratified detection, resulting in a 4.2% increase in detection accuracy. This improvement is achieved by automatically selecting appropriate Anchor Box sizes based on the distribution of Ambrosia trifida in the dataset and generating multi-scale Anchor Boxes at different levels. The detection results after employing k-Means-based Anchor Box stratified detection are shown in **Figures 13A**, **B**. In the figures, the orange rectangles represent the ground truth positions of Ambrosia trifida, the blue rectangles represent the detected positions, and the red rectangles represent falsely detected positions. It can be observed that the number of missed Ambrosia trifida instances reduced from 19 to 7 after using k-Means-based Anchor Box stratified detection, demonstrating a significant improvement in performance.

The improved loss function resulted in a 1.7% increase in detection accuracy for YOLOv5-KE compared to YOLOv5. This improved loss function biases the regression process towards high-quality anchor boxes, reducing the negative impact of low-quality anchor boxes on the recognition process. This allows the network to learn enough high-quality positive anchor boxes and improve its ability to detect small Ambrosia trifida. By minimizing the EIOU loss function, the model can better fit the target bounding boxes, thereby improving object detection accuracy.

Improvement of detection box fusion algorithm resulted in a 3.4% increase in detection accuracy for YOLOv5-KE compared to YOLOv5. This is because the feature information of Ambrosia trifida in UAV images is relatively weak under the conditions of partial overlap and occlusion of leaves, the NMS algorithm of YOLOv5 is prone to large errors for overlapping pre-selected boxes, which may make it difficult for a single-scale detection method to accurately detect small targets. The WBS algorithm of YOLOv5-KE can fuse detection results from multiple scales by considering multiple factors and weighted averaging, thereby reducing this error to some extent and improving the robustness of object detection results, resulting in a higher detection rate and accuracy for small targets.

Input resolution is an important factor affecting image detection accuracy. Higher resolution leads to higher detection accuracy. If the input image resolution was too low, the model will not be able to recognize significant features that aid image recognition. In this study, when the input image resolution was 640×640, the detection accuracy reached its highest level, consistent with previous research findings and universal rules (Wang Q. et al., 2022).

The height at which UAV images are taken also affects recognition accuracy. In this study, images of Ambrosia trifida were captured using a UAV at flight hights of 5m, 10m, and 15m, and the accuracy of recognizing Ambrosia trifida was decreasing as the flight altitude increased. When UAV fly at the height of 15m, Ambrosia trifida becomes too small in images, making them prone to being missed. Previous studies have confirmed that with the increase of drone flying height, the target in the image becomes smaller, reducing the information of the target object in the image, leading to a lower recognition accuracy (Liu et al., 2023). However, it is worth noting that if the drone flying height is too low, it can also cause image distortion and affect recognition accuracy (Xiong et al., 2023). In this study, when the UAV flying at a height of 5m, the detail data of clover ragweed in the image were kept better, it will not cause image distortion, so that the small target Ambrosia trifida can be detected more easily.

## 5 Conclusions

Ambrosia trifida is a malignant weed and was one of the first invasive species to be listed in the “List of Alien Invasive Species in China”. Utilizing UAV images for Ambrosia trifida monitoring is an effective measure to control its growth. However, the small size, high density, and overlapping features of Ambrosia trifida in UAV images result in low detection accuracy. In this study, a YOLOv5-KE UAV image detection algorithm was proposed to detect Ambrosia trifida. Experimental results show that YOLOv5-KE achieves a detection accuracy of 93.9%, making it an effective algorithm for detecting Ambrosia trifida under complex field conditions. The analysis of YOLOv5-KE also demonstrates its potential for detecting other types of weed with small size, high density, and overlapping leaves.

## Data availability statement

The raw data supporting the conclusions of this article will be made available by the authors, without undue reservation.

## Author contributions

CX: Project administration, Resources, Writing – review & editing. CT: Writing – original draft, Writing – review & editing. MH: Software, Writing – review & editing. ZZ: Investigation, Writing – review & editing. WD: Validation, Writing – review & editing. SJ: Data curation, Writing – review & editing. WJ: Funding acquisition, Validation, Writing – review & editing.
